# Generalized Linear Model for Mapping Discrete Trait Loci Implemented with LASSO Algorithm

**DOI:** 10.1371/journal.pone.0106985

**Published:** 2014-09-11

**Authors:** Jun Xing, Huijiang Gao, Yang Wu, Yani Wu, Hongwang Li, Runqing Yang

**Affiliations:** 1 Department of Gastroenterology, Tumor Hospital of Harbin Medical University, Harbin, People's Republic of China; 2 Institute of Animal Science, Chinese Academy of Agricultural Science, Beijing, People's Republic of China; 3 School of Agriculture and Biology, Shanghai Jiaotong University, Shanghai, People's Republic of China; 4 Research Centre for Fisheries Resource and Environment, Chinese Academy of Fishery Sciences, Beijing, People's Republic of China; Pennsylvania State University, United States of America

## Abstract

Generalized estimating equation (GEE) algorithm under a heterogeneous residual variance model is an extension of the iteratively reweighted least squares (IRLS) method for continuous traits to discrete traits. In contrast to mixture model-based expectation–maximization (EM) algorithm, the GEE algorithm can well detect quantitative trait locus (QTL), especially large effect QTLs located in large marker intervals in the manner of high computing speed. Based on a single QTL model, however, the GEE algorithm has very limited statistical power to detect multiple QTLs because of ignoring other linked QTLs. In this study, the fast least absolute shrinkage and selection operator (LASSO) is derived for generalized linear model (GLM) with all possible link functions. Under a heterogeneous residual variance model, the LASSO for GLM is used to iteratively estimate the non-zero genetic effects of those loci over entire genome. The iteratively reweighted LASSO is therefore extended to mapping QTL for discrete traits, such as ordinal, binary, and Poisson traits. The simulated and real data analyses are conducted to demonstrate the efficiency of the proposed method to simultaneously identify multiple QTLs for binary and Poisson traits as examples.

## Introduction

Corresponding to continuous and discrete random variables in statistics, quantitative traits are classified into continuous and discrete traits in quantitative genetics. In contrast to discrete traits, continuous traits especially normally distributed ones are analyzed by taking advantage of the extensively developed inference methods available for linear models. Actually, mapping methods for continuous quantitative traits are developed prior to discrete traits. The earliest QTL mapping for continuous traits can be traced back to the interval mapping developed by Lander and Botstein [1], while the first group of people to map ordinal traits using the EM algorithm is credited to Hackett and Weller [2] and Xu and Atchley [3].

Binary and categorical discrete traits are commonly observed that typically follow binomial and multinomial distributions. A binary trait including only two categories is a special case of categorical or ordinal trait. Also, binomial trait and multinomial trait can be regarded as the derivatives of binary and categorical or ordinal traits, defined by the proportions of the number of events happened among the total number of trials. Traits measured as counts are often called Poisson traits because they are usually modeled by a Poisson distribution. The generalized linear model (GLM) therefore becomes a natural choice for analyzing the discrete traits with the above mentioned distributions [4], [5]. Some applications of GLM to mapping QTLs have been conducted for binary traits [3], [6], [7], ordinal traits [2], [8] and Poisson traits [9], [10]. The IRLS for the normally distributed traits was extended to analyze binary traits [11]–[13], which greatly improves the computational efficiency with little loss in power. Especially, the EM algorithm within the framework of GLM have been developed to simplify QTL mapping for binary traits and ordinal traits [14], [15]. In addition, the GEE approach for GLM has been adopted to comprehensively analyze multiple mixed traits of continuous and discrete trait components [16].

Based on the interval mapping with GLM models, a set of mapping methods [7], [17]–[19] have been developed to simultaneously map multiple QTLs for discrete traits. As an alternative, Bayesian mapping method with reversible-jump MCMC sampling [20] was proposed to infer QTLs for binary traits [7]. Subsequently, Yi et al. [18] applied a stochastic search variable selection method for Bayesian mapping of ordinal traits which remarkably improved the sampling efficiency for model parameters. By fitting a continuous prior distribution on genetic effects, most recently, hierarchical generalized linear models and computationally efficient algorithms have been further developed for genome-wide analysis of QTL for various types of phenotypes in experimental crosses [19]. In Bayesian mapping, only the method with reversible-jump MCMC sampling belongs to fully Bayesian. However, the reversible-jump MCMC sampling is usually subject to poor mixing. Some Bayesian methods use imputed QTL genotypes based on the conditional expectations of the genotype given on the flanking marker information, but ignores the uncertainties of the imputation process. Unfortunately, no efficient and convincing method for statistical inference of QTLs is provided.

As an extension of the IRLS method for continuous traits to discrete traits, GEE algorithm under a heterogeneous residual variance model can well characterize QTLs, especially large QTLs located in large marker intervals, in the manner of high computing speed [21]. Such a method has been developed for the interval mapping of discrete traits. Thus, it has a limited statistical power to identify multiple QTLs, without considering other linked QTLs. The objectives of this study is to derive a penalized generalized linear model with LASSO penalty, and then, to iteratively estimate the non-zero genetic effects of those loci over the entire genome, under a heterogeneous residual variance model. The iteratively reweighted LASSO algorithm [22] is extended to QTL mapping for discrete traits such as ordinal, binary, and Poisson traits.

## Methods

### Generalized linear genetic model

In a QTL mapping analysis, a genetically designed population is required to construct linkage relationship between putative QTL and markers. In such a population, *n* individuals are observed for phenotypic values and are genotyped for reasonably dense co-dominant markers. A genetic linkage map can be constructed based on the observed markers or can be obtained from prior studies. Based on the linkage information, QTLs are identified by inferring the significance of genetic effects for loci on or between markers.

If the trait of interest is normally distributed, the effects of these loci on phenotype are generally represented by the following linear model:
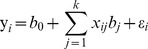
(1)where 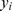 for 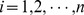 is the phenotypic value of the normal trait; 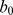 is the population mean; *k* is the number of putative loci; 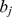
is the genetic effect at the *j*th locus; 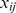 is the indicator variable determined by the genotypes of the *j*th locus. Note that only of the additive genetic effect is considered here for the simplification of description, which is suitable for backcross, double haploid and recombinant inbred lines.

In model (1), the residual error 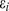 is assumed to follow a normal distribution with 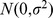. Hence, the expectation of a normal phenotype has a linear predictor:
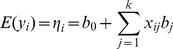
(2)


In many situations, however, a trait in question are measured in discrete form, including binary, binomial, categorical, multinomial and Poisson traits. Their distributions, summarized in Table 1, belong to an exponential distribution family with different link functions. The relationship between the mean of a discrete phenotype (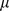) and the linear predictor of genetic effects of *k* loci (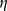) is formulated by means of a link function, denoted by
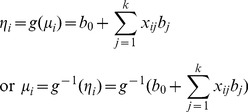
(3)for 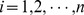.

**Table 1:** The commonly used distributions in the GLM for discrete traits.

Distribution	Link name	Link function	Mean function	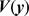	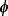
Normal	Identity	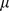	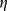	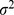	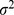
Poisson	Log	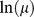	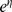	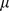	1
	Identity	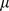	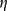		
	Sqrt	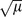	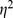		
Binomial	Logit	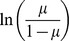	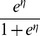	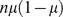	1
	Cloglog	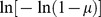	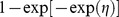		
	Probit	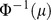	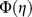		
	Log	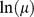	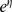		
Multinomial	As above	As above	As above	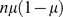	1

This is the GLM for multiple QTL mapping with discrete traits. In the model, *g* is the link function. Moreover, the variance of discrete phenotype 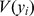 can be derived from the distribution of each discrete trait (Also shown in Table 1), which is useful for estimating model parameters.

### Genetic effect estimation

Theoretically speaking, the reweighted least square method by Wedderburn [5] can be used to estimate the parameters in model (3). But implementation of this method is not straightforward, due to the fact that the number of parameters estimated may be far greater than sample size and the values of indicator variables are missing at loci between markers. According to the least squares method of Haley and Knott [23], the missing values of indicator variables can be simply replaced by its expectation of conditional probability given on flanking marker genotypes. This replacement, however, could result in over dispersion (Xu 1998). From the linear predictor in model (3), the over-dispersion is calculated as
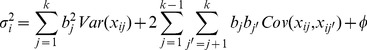
(4)where 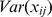 and 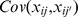 depend on genetically designed population, as derived in Liu et al. [22]; 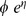 is the parameter of dispersion in an exponential family. To adjust for the heterogeneity of over-dispersion [21], the linear predictor is standardized by the over-dispersion parameter, which lead to



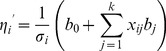
(5)Instead of 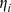, the standardized linear predictor 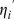 is substituted into model (3). As in the reweighted least square method for a generalized linear model, it is defined that
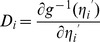
(6)

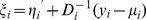
(7)

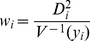
(8)


By Taylor expansion, the log-likelihood about model parameters is quadratically approximated by
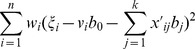
(9)with 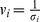 and 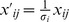.

With more model parameters than sample size, the unique estimates of genetic effects can not be obtained by minimizing the log-likelihood above. Actually, there are few non-zero and significant genetic effects in model (3), because the number of QTLs for a trait is generally not large. In this case, the LASSO penalized method with a coordinate descent step can efficiently shrink most of genetic effects to zeros by minimizing the following function [24], [25]:
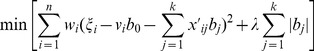
(10)where 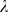 is a tuning parameter which will be chosen by cross validation.

In solving model parameters with LASSO, iterations are required, as response variable 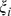, independent variables 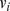 and 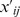 as well as weighted value 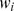 are all a function of the estimated parameters. Without heterogeneous over-dispersion, the software R/glmnet can be applied to efficiently search for sparse solutions in the oversaturated GLM model [25]. Taking the glmnet as the inner loop, the iterative procedure is implemented in the following steps:

1. Initialize 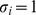 and all genetic effects as zeros
2. Shrink genetic effects with the unweighted glmnet
3. Update 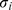 using non-zero genetic effects
4. Shrink genetic effects with the iteratively weighted glmnet
5. Repeat step 2 and 4 until certain convergence criteria are satisfied.

### Statistical inference for QTLs

The LASSO for oversaturated GLM can provide estimates of non-zero genetic effects, but cannot do significance test for the estimates. After shrinkage estimation, the number of non-zero effects is generally less than the sample size. Substituting for the glmnet in iterative procedure, therefore, the reweighted least squares method for common GLM can be employed to estimate non-zero genetic effects:
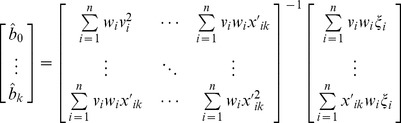
(11)


The variance-covariance matrix of the model parameters is estimated by
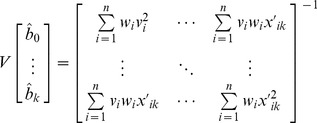
(12)


The *t* test statistic is used to infer the significance of the non-zero effects, which is calculated as 
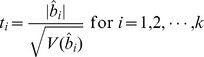
(13)


It needs to be specially noticed that genetic effects re-estimated by reweighted least square method may be biased upward due to high variable selection of LASSO above. Meanwhile, population structure and marker density influence distribution of the *t* test statistic still. Permutation tests [26] is therefore introduced to adjust the critical value of *t* test statistic. The loci corresponding to significant genetic Meanwhile, population structure and mark effects are determined as the QTLs for trait of interest.

### Simulations

The purpose of simulation is to demonstrate the efficiency of the method proposed here (IRglmnet for short), by comparing it to the GEE (IRGEE for short) algorithm under a heterogeneous residual variance model and unweighted glmnet (UWglmnet for short). Six chromosomes, each of length 100 cM with 11 evenly placed co-dominant markers are simulated in a backcross population with a sample size of 200 and 400. Total 10 QTLs are simulated on the 6 chromosomes, whose positions and genetic effects are listed in Table 2 and Table 3. Assuming population mean to be zero, the expectation 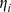 is calculated based on the simulated genotypes of the QTLs and the expectation 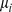 of discrete traits based on the link function. Taking binary and Poisson traits as examples, phenotypic values are randomly generated from binomial and Poisson distributions with their known expectations.

**Table 2:** Mean estimates and standard deviations (in parentheses) of QTL positions detected with three mapping methods for the simulated datasets.

Trait	Sample size	QTL no.	Q_1_	Q_2_	Q_3_	Q_4_	Q_5_	Q_6_	Q_7_	Q_8_	Q_9_	Q_10_
		True position	23	56	148	193	267	332	390	478	522	574
Binary	200	IRglmnet	23.2(2.5)	57.2(2.3)	150.3 (2.8)	194.3(2.6)	271.7(2.7)	335.0(2.4)	393.5(2.3)	480.3(3.7)	530.0(3.2)	578.6(2.8)
		UWglmnet	23.1 (2.7)	56.9(2.3)	150.0(2.7)	194.6(2.7)	271.9(2.8)	334.6(2.4)	393.8(2.3)	480.7(3.5)	526.5(3.2)	579.0(3.0)
		IRGEE	28.9(5.5)	55.0(4.5)	154.6(7.4)	191.4(6.1)	276.5(16.2)	335.8(8.1)	389.0(7.0)	481.5(7.5)	545.0(4.4)	586.0(8.8)
	400	IRglmnet	23.7(2.1)	57.1(1.5)	149.8(2.8)	194.7(2.6)	270.9(2.6)	335.1(2.5)	393.8(2.1)	480.4(2.7)	526.8(1.7)	578.9(2.8)
		UWglmnet	23.4(2.2)	57.0(1.5)	149.6(3.0)	194.7(2.5)	271.1 (2.8)	335.0(2.5)	393.8(2.3)	480.2(2.8)	526.5(2.9)	579.0(2.9)
		IRGEE	29.1(4.8)	54.5(3.5)	155.8(7.8)	191.4 (6.5)	278.7(7.8)	333.9(6.2)	398.7(6.7)	480.8(5.8)	546.0(7.9)	581.9(7.0)
Poisson	200	IRglmnet	23.8(2.6)	56.5(2.5)	149.9(2.8)	194.5(2.5)	271.7(2.8)	335.1(2.6)	393.8 (2.2)	480.9(2.9)	526.4(2.5)	578.8(2.6)
		UWglmnet	23.7(2.5)	56.1(2.4)	150.1 (2.6)	194.5(2.7)	270.84(2.8)	335.1(2.7)	393.5(2.4)	480.7(2.8)	526.6(2.4)	578.9(2.8)
		IRGEE	16.9(8.7)	52.7(9.4)	152.1(9.3)	191.2(8.2)	266.6(12.3)	335.8(10.9)	395.4(11.0)	481.5(8.2)	524.5611.6)	581.1(10.0)
	400	IRglmnet	24.22(2.3)	56.2(2.3)	150.1(2.5)	194.7(2.5)	270.8(2.9)	335.2(2.5)	393.8(2.3)	480.8(2.3)	527.0(2.7)	579.4 (2.6)
		UWglmnet	24.2(2.3)	56.1(2.3)	150.1(2.5)	194.6(2.5)	270.4 (3.0)	335.1(2.5)	394.1(2.1)	480.8(2.5	526.62(2.4)	579.6(2.5)
		IRGEE	20.2(10.8)	49.3(7.4)	154.3(7.3)	192.1(6.9)	269.4(12.0)	333.7(6.7)	396.1(7.2)	480.4(7.8)	524.8(9.4)	580.0 (6.4)

**Table 3:** Mean estimates and standard deviations (in parentheses) of QTL effects obtained with three mapping methods for the simulated datasets.

Trait	Sample size	QTL no.	Q_1_	Q_ 2_	Q_ 3_	Q_4_	Q_ 5_	Q_ 6_	Q_ 7_	Q_ 8_	Q_ 9_	Q_ 10_
		True effect	1.5	2.0	0.72	1.1	−0.22	0.70	−0.65	1.25	0.35	−0.80
Binary	200	IRglmnet	1.51(0.26)	1.88(0.34)	0.97(0.18)	1.25(0.25)	−0.40(0.11)	0.91(0.19)	−0.89(0.19)	1.18(0.28)	0.60(0.18)	−0.96(0.21)
		UWglmnet	1.25(0.35)	1.59(0.42)	0.83(0.19)	1.02(0.28)	−0.31(0.10)	0.8(0.19)	−0.8(0.19)	0.98(0.29)	0.59(0.18)	−0.80(0.24)
		IRGEE	0.78(0.39)	0.90(0.38)	0.38(0.18)	0.41(0.17)	−0.33(0.11)	0.34(0.24)	−0.33(0.11)	0.37(0.26)	−0.3(0.21)	−0.36(0.22)
	400	IRglmnet	1.59(0.27)	1.99(0.34)	0.83(0.21)	1.13(0.31)	−0.35(0.08)	0.77(0.20)	−0.75(0.20)	1.23(0.32)	0.56(0.07)	−0.82(0.21)
		UWglmnet	1.17(0.31)	1.49(0.23)	0.60(0.14)	0.79(0.17)	−0.30(0.09)	0.57(0.13)	−0.55(0.12)	0.89(0.18)	0.46(0.07)	−0.61(0.14)
		IRGEE	0.76(0.17)	0.89(0.17)	0.33(0.16)	0.36(0.17)	−0.25(0.13)	0.25(0.14)	−0.15(0.19)	0.32(0.16)	−0.22(0.13)	−0.26(0.13)
Poisson	200	IRglmnet	1.38(0.31)	1.70(0.35)	0.80(0.18)	1.07(0.17)	−0.23(0.20)	0.76(0.21)	−0.73(0. 21)	1.28(0.25)	0.35(0.16)	−0.81(0.16)
		UWglmnet	1.41(0.34)	1.64(0.43)	0.73(0.19)	1.03(0.17)	−0.23(0.25)	0.67(0.27)	−0.64(0.27)	1.19(0.26)	0.35(0.17)	−0.75(0.17)
		IRGEE	1.27(0.23)	1.21(0.21)	1.14(0.21)	1.20(0.29)	−0.25(0.50)	0.53(0.37)	−0.33(0.57)	1.13(0.17)	0.19(0.52)	−0.66(0.39)
	400	IRglmnet	1.34(0.20)	1.61(0.23)	0.76(0.11)	1.07(0.12)	−0.24(0.09)	0.77(0.14)	−0.74(0.15)	1.24(0.13)	0.39(0.11)	−0.85(0.15)
		UWglmnet	1.42(0.28)	1.49(0.34)	0.72(0.14)	1.02(0.14)	−0.22(0.11)	0.69(0.15)	−0.65(0.17)	1.17(0.13)	0.35(0.11)	−0.76(0.16)
		IRGEE	1.21(0.21)	1.21(0.21)	1.08(0.17)	1.17(0.16)	−0.4(0.22)	0.51(0.19)	−0.43(0.34)	1.1(0.15)	0.17(0.27)	−0.73(0.20)

### Alopecia areata in mouse

To locate QTLs linked with alopecia areata, an F_2_ population was generated from crossing the strain of C3H/HeJ and C57BL/6J mice [27]. The 138 alopecia areata and 214 clinically normal mice were genotyped at 12 months of age using 211 microsatellite markers. Linkage maps and marker positions were reported on the website www.informatics.jax.org.

### Tiller numbers in rice

This is a Poisson dataset for mapping QTL of tiller numbers in rice [28]. A doubled-haploid (DH) population of 123 lines was derived from the cross between two inbred lines, semidwarf IR64 and tall Azucena [29]. Based on this population, a genetic linkage map of 2005 cM long was constructed using 175 genetic markers. For the 123 DH lines, each containing five plants, tiller numbers were observed every 10 days until all lines had headed. Phenotypic value of each line was obtained by averaging over five plants.

## Results

### Simulation study

The simulated datasets are analyzed by using IRglmnet, UWglmnet and IRGEE. For convenience to compare the three mapping methods, all test statistics are transformed to -log(*p*), where *p* is the probability of greater than the realized statistic values. The critical values of the test statistic are determined through simulating 1000 samples under the null model with zero genetic effects. They are slightly distinguishable among the three mapping methods and two sample sizes (Results not shown). The simulations are replicated 500 times for estimating QTL parameters and assessing the statistical power of QTL detection. Statistical power of QTL detection is counted by each locus as the percentage of the number of those simulations that statistic value exceeds critical value at the locus.

The statistical performances of different scenarios are presented in Table 2 for position estimate comparison, in Table 3 for QTL parameter estimate comparison and in Table 4 for power comparison, As can be seen, the IRglmnet is mostly identical to the UWglmnet and the two glmnet methods are advantageous to IRGEE in terms of power to detect QTL. The IRglmnet methods can accurately estimate QTL genetic effects, while UWglmnet somewhat underestimates and IRGEE can not estimate well QTL effects. Meanwhile, both glmnets are able to detect QTL positions with higher precision than IRGEE. Under the same genetic design and sample size, the two traits analyzed are evidently distinct in QTL parameter estimation and power of QTL detection at the QTLs of small genetic effects. As compared to normally distributed traits in Liu et al. [22], the statistical powers to detect QTL for the two analyzed traits are higher at all the simulated QTLs except for the QTLs of small genetic effects, even with large sample size. This implies that in principle, it is generally difficult to detect QTLs for discrete traits. In addition, the statistical performance of each mapping method increases as sample size and QTL genetic effect increase, as observed in usual QTL mapping.

**Table 4:** Statistical powers of QTL detection obtained with three mapping methods for the simulated datasets.

Trait	Sample size	Chr. no.	C_1_		C_2_		C_3_	C_4_		C_5_	C_6_	
		QTL no.	Q_1_	Q_ 2_	Q_3_	Q_4_	Q_5_	Q_6_	Q_7_	Q_8_	Q_9_	Q_10_
Binary	200	IRglmnet	83.0	85.7	27.5	49.9	5.5	24.2	17.9	71.0	5.3	26.6
		UWglmnet	83.6	87.1	27.1	49.2	5.2	23.6	23.0	78.8	5.9	30.2
		IRGEE	68.1	88.0	27.2	32.3	2.2	6.3	2.3	47.8	3.1	11.5
	400	IRglmnet	92.9	91.8	57.2	82.1	7.3	60.5	59.8	84.9	11.8	69.9
		UWglmnet	93.1	95.1	60.0	78.5	7.5	62.8	63.4	85.8	12.4	65.9
		IRGEE	74.2	92.2	56.0	80.8	3.2	17.0	15.4	79.4	5.2	20.4
Poisson	200	IRglmnet	83.6	82.5	74.2	81.3	38.6	76.5	83.4	83.4	57.8	70.6
		UWglmnet	83.2	82.4	74.3	83.8	40.5	79.6	83.0	78.6	56.5	73.9
		IRGEE	80.1	78.2	70.4	74.8	23.0	65.5	62.3	76.2	30.2	54.3
	400	IRglmnet	87.7	96.2	82.9	91.9	59.0	88.8	91.4	91.2	69.8	79.8
		UWglmnet	88.8	90.4	78.4	91.6	56.2	86.8	91.6	89.0	71.0	80.8
		IRGEE	78.7	86.3	80.5	84.3	30.4	82.2	82.6	88.2	48.3	68.2

The advantage of our proposed method lies in the computing efficiency as well. Although the IRglmnet method with cross validation takes more computing time than the UWglmnet, the two glmnet methods run considerably fast, as compared to the IRGEE. On an Intel core 4 PC with a 3.8 GHz processor, the UWglmnet and IRglmnet for binary data consume 10.8 seconds and 24.5 seconds on average, respectively, whereas the IRGEE takes 1.2 minutes under a sample size of 200. For Poisson data, the UWglmnet, IRglmnet and IRGEE run 14.3 seconds, 33.6 seconds and 1.8 minutes, respectively. The difference in computing time-consuming gets larger as the sample size increases.

### Mapping QTL for alopecia areata

In an F_2_ population, there are three genotypes at each locus, denoted by QQ, Qq and qq, so that QTL genetic effect can be partitioned into additive and dominance effects. The linear predictor for alopecia areata traits is described as 
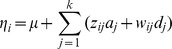
where, 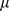 is the population mean, 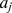 is the additive effect at the *j*th locus, 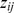 is the indicator variable corresponding to the additive effect, defined as +1 for QQ, 0 for Qq and −1 for qq. 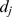 is the dominance effect at the *j*th locus, 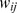 is the indicator variable corresponding to the dominance effect, defined as 0 for homozygote and 1 for heterozygote.

**Figure 1:**
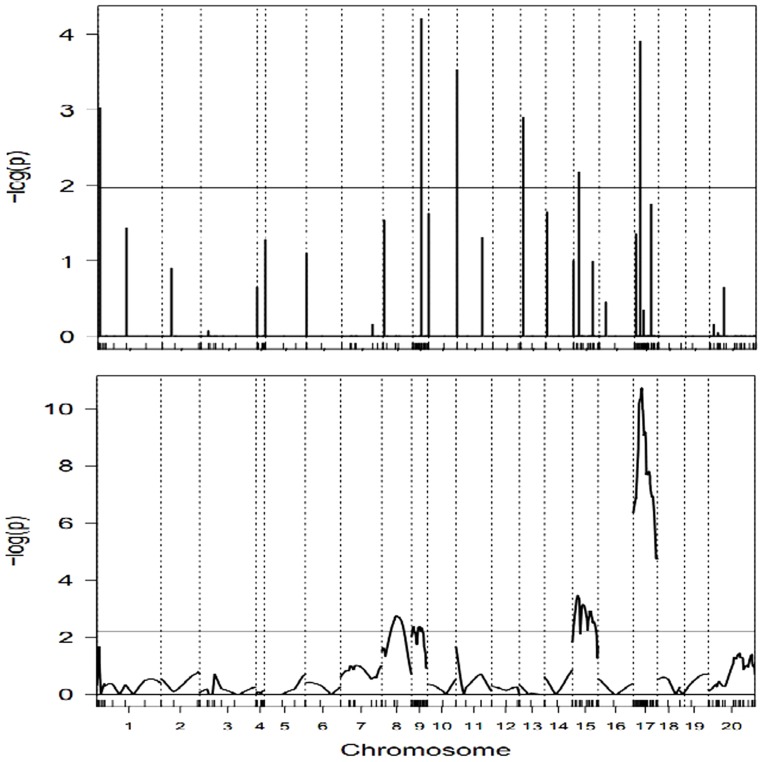
The profiles of -log(p) test statistics of additive genetic effects obtained with IRglmnet method (upper panel) and IRGEE method (lower panel) for alopecia areata. In each plot, the genome-wide critical value is marked by a horizontal reference line. Chromosomes are separated by the vertical dotted lines and marker positions are indicated by the ticks on the horizontal axis.

**Figure 2:**
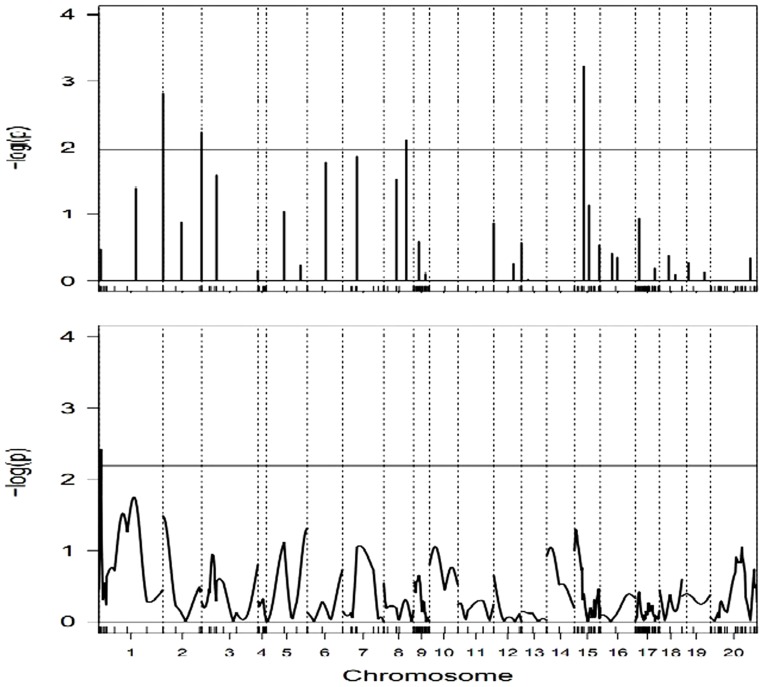
The profiles of -log(p) test statistics of dominance genetic effects obtained with IRglmnet method (upper panel) and IRGEE method (lower panel) for alopecia areata. In each plot, the genome-wide critical value is marked by a horizontal reference line. Chromosomes are separated by the vertical dotted lines and marker positions are indicated by the ticks on the horizontal axis.

With probit link function, the dataset is analyzed by using IRglmnet, UWglmnet and IRGEE methods. The genome-wide critical threshold values for declaring QTL significance are obtained by using 1000 permutation tests. The critical values are distinguishable between the two glmnets methods and IRGEE method, which is marked by horizontal reference line in Figure 1 and Figure 2. The comparative plots in the profiles of –log(p) statistics between IRglmnet and IRGEE methods are depicted in Figure 1 and Figure 2 by the mode of inheritance. The overdispersion parameter for each individual is much closed to 1 in running IRglmnet method, so that the results obtained with the UWglmnet are exactly the same as those obtained with the IRglmnet. The QTLs are generally determined according to the peaks that exceed corresponding critical values. As can be seen from the two Figures, the IRglmnet finds not only those QTLs detected with IRGEE but also more QTLs than those detected with IRGEE. Surprisingly, most of QTLs are located on markers in the genetic map with moderate density.

Table 5 tabulates parameter estimates of QTLs detected with three mapping methods. A total of 10 QTLs are identified for alopecia areata, of which, 6 are inherited in the additive mode and 4 in the dominance mode. Interestingly, the QTLs on chromosome 1 and chromosome 8 are completely different in the mode of inheritance between the two glmnet methods and IRGEE method. The proportions of phenotypic variation explained by the detectable QTLs varied from 2% to 49%. The largest heritability (49%) is of the QTL on chromosome 17, which is more than five times of the second highest heritability (9%). The estimates for genetic effects from the IRglmnet are twice more than those from the UWglmnet, but their estimated heritabilies are roughly the same, except for the largest heritability. Meanwhile, the three mapping methods are able to consistently detect major locus on mouse chromosome 17 and minor locus on chromosome 9, as reported in Sundberg et al. [27].

**Table 5:** Estimated QTL parameters obtained with the three mapping methods for alopecia areata in an F_2_ mouse population.

Inheritance Mode	QTL No.	IRglmnet					UWglmnet		IRGEE	
		Chr-pos. (cM)	Marker interval	Effect	Heritability	-log(P)	Effect	Heritability	Chr-pos. (cM)	Effect
Additive	1	1–3	D1Mit231	−1.05	0.03	3.02	−0.37	0.02	8–22	−0.35
	2	9–13	D9Mit162	1.31	0.05	4.21	0.53	0.04	9–3	0.28
	3	10–43	D10Mit180	1.05	0.03	3.53	0.50	0.04		
	4	13–4	D13Mit179∼D13Mit159	1.21	0.04	2.90	0.57	0.05		
	5	15–8	D15Mit115∼D15Mit270	−1.14	0.04	2.19	−0.49	0.03	15–8	−0.39
	6	17–8.8	D17Mit80	−4.00	0.49	3.91	−1.44	0.30	17–12.8	−0.75
Dominance	1	2–0	D2Mit237	1.59	0.04	2.81	0.71	0.04	1–3	−0.41
	2	2–58.3	D2Mit456	−1.27	0.02	2.23	−0.62	0.03		
	3	8–34	D8Mit75∼D8Mit167	−2.48	0.09	2.10	−1.20	0.10		
	4	15–14.2	D15Mit209	2.45	0.09	3.23	1.02	0.07		

### Mapping QTL for tiller numbers

**Figure 3:**
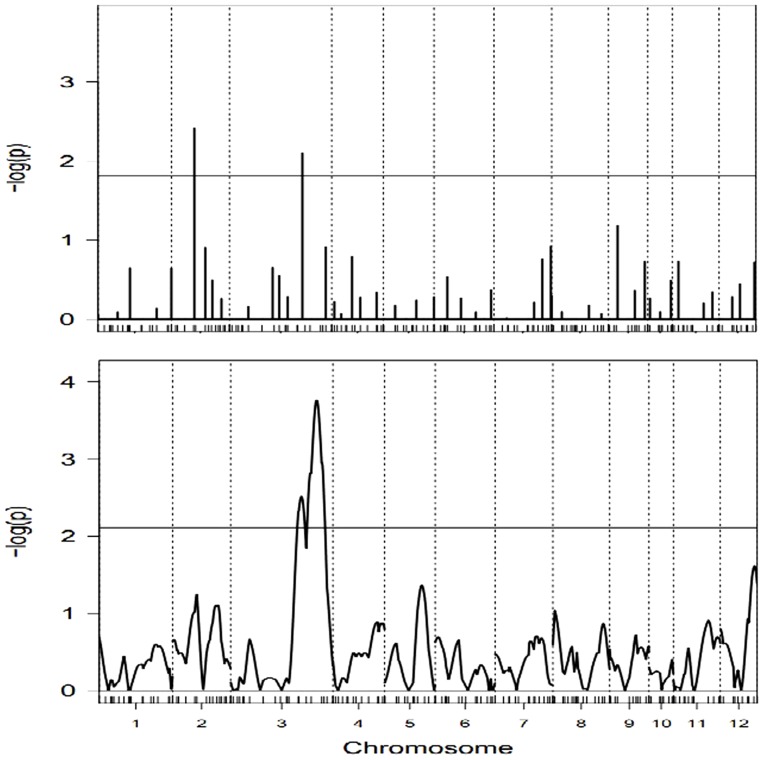
The profiles of -log(p) test statistics obtained with IRglmnet method (upper panel) and IRGEE method (lower panel) for tiller numbers. In each plot, the genome-wide critical value is marked by a horizontal reference line. Chromosomes are separated by the vertical dotted lines and marker positions are indicated by the ticks on the horizontal axis.

Taking the tiller numbers at the third stage (30 days after transplanting) as an example, the QTLs for the traits are located by using the three competing mapping methods. Figure 3 (upper panel) illustrates that there are two peaks that pass through the horizontal line of critical value 2.043 at 5% genome-wide significant level. This suggests that the two QTLs are indentified by the IRglmnet and the UWglmnet. One of the detected QTLs is located between markers MK23 and MK24 on chromosome 2, another between markers MK48 and MK49 on chromosome 3. They explain 1.9% and 2.1% of phenotypic variance, respectively. There is almost no difference in the estimated heritability between the UWglmnet and the IRglmnet, but the estimates for genetic effect obtained with UWglmnet are less than that with IRglmnet. In contrast, the IRGEE finds only one QTL of those identified by both glmnet methods, as shown in Figure 3 (lower panel).

## Discussion

Two extensions are realized to map QTL for discrete traits: one is that of the GEE algorithm under a heterogeneous residual variance model by Xu and Hu [21] for a single QTL model to multiple QTL model, and another is that of IRLASSO for the continuous normal traits [22] to discrete ones. The glmnet with coordinate descent step is used for fast estimation of non-zero effects, followed by few non-zero genetic effects estimated and statistically inferred with a regular GLM. Like regular interval mapping, the method proposed here can, not only estimate QTL effects, but also assess the significance of QTLs. Although our mapping method is developed for improving linkage analysis with low marker density, it is also appropriate for missing genotypes that always happen in QTL mapping.

R/glmnet can efficiently fit binary, categorical and Poisson data with logistic and Poisson regression models [22]. However, it can not be used to directly analyze binomial and multinomial data and only logit and log link functions are used in solving for the oversaturated GLM. In this study, a general LASSO procedure for the GLM is derived based on all possible link function for discrete traits, which can be incorporated into the R/glmnet with little modification. From a statistical viewpoint, the choice for link function can be somewhat arbitrary, but it is necessary to understand the biological meaning of discrete traits. For instance, the biological mechanism of binary and categorical traits can be well interpreted by the threshold model with the probit link function.

Our proposed method can be simplified to a notable extent in the practice of QTL mapping. As demonstrated in simulations, the UWglmnet is mostly consistent with IRglmnet method in terms of power to detect QTL, although it underestimates the genetic effects of QTLs. By removing the iteratively weighted step for IRglmnet, non-zero effects can be solved by using UWglmnet for simple computation. The iteratively weighted step is only used in GLM analysis for reestimating few non-zero genetic effects. In R/glmnet, additionally, cross validation (CV) is always introduced to improve shrinking efficiency. The fewer non-zero genetic effects are retained after the CV.glmnet, but more computing time is needed. Actually, the CV step can be ignored in the QTL mapping, as the CV.glmnet just drops the redundant non-zero genetic effects of those obtained with the glmnet without CV. Prior to the GLM analysis, the redundant non-zero genetic effects solved by using the glmnet without CV can be simply removed by restricting that there is only one QTL within a marker interval. Without the iteratively weighted step and cross-validation for glmnet, our proposed mapping method can save more computing time than the GEE algorithm under a heterogeneous residual variance model by Xu and Hu [21]. Our proposed method has been coded in the program on the IRLASSO [22], which can handle normally distributed, binary, binomial, ordinal, multinomial and Poisson traits. The program is freely available upon request from the authors.
